# Reconstructing networks of pathways via significance analysis of their intersections

**DOI:** 10.1186/1471-2105-9-S4-S9

**Published:** 2008-04-25

**Authors:** Mirko Francesconi, Daniel Remondini, Nicola Neretti, John M Sedivy, Leon N Cooper, Ettore Verondini, Luciano Milanesi, Gastone Castellani

**Affiliations:** 1 Centro Interdipartimentale “L. Galvani”, Università di Bologna, , Bologna 40127, Italy; 2 Dipartimento di Morfofisiologia veterinaria e Produzioni Animali (DIMORFIPA), Università di Bologna, Bologna 40064, Italy; 3 Department of Physics , Università di Bologna, Bologna 40127, Italy; 4 Institute for Brain and Neural Systems, Brown University, Providence RI 02906, USA; 5 Department of Molecular Biology, Cell Biology and Biochemistry, Brown University, Providence, RI 02903, USA; 6 Department of Physics, Brown University, Providence RI 02906, USA; 7 Istituto di Tecnologie Biomediche (ITB) CNR, Milano 20090, Italy

## Abstract

**Background:**

Significance analysis at single gene level may suffer from the limited number of samples and experimental noise that can severely limit the power of the chosen statistical test. This problem is typically approached by applying post hoc corrections to control the false discovery rate, without taking into account prior biological knowledge. Pathway or gene ontology analysis can provide an alternative way to relax the significance threshold applied to single genes and may lead to a better biological interpretation.

**Results:**

Here we propose a new analysis method based on the study of networks of pathways. These networks are reconstructed considering both the significance of single pathways (network nodes) and the intersection between them (links).

We apply this method for the reconstruction of networks of pathways to two gene expression datasets: the first one obtained from a c-Myc rat fibroblast cell line expressing a conditional Myc-estrogen receptor oncoprotein; the second one obtained from the comparison of Acute Myeloid Leukemia and Acute Lymphoblastic Leukemia derived from bone marrow samples.

**Conclusion:**

Our method extends statistical models that have been recently adopted for the significance analysis of functional groups of genes to infer links between these groups. We show that groups of genes at the interface between different pathways can be considered as relevant even if the pathways they belong to are not significant by themselves.

## Background

High-throughput gene expression analysis has become one of the methods of choice in the exploratory phase of cellular molecular biology and medical research studies. Although microarray technology has improved measurement accuracy, and new statistical algorithms for better signal estimation have been developed [1-3], reproducibility remains an issue [4]. A way to overcome this difficulty is to move the analysis from the gene level to a higher level where genes are grouped into functional categories. This approach has been shown to be more robust and reproducible [5,6], and leads to an easier biological interpretation of the experimental observations.

Gene Ontology (GO) [7] and pathways are the two main gene-grouping schemes in use. GO organizes genes according to a hierarchy of terms divided into three categories: “cellular component”, “biological process”, and “molecular function”. Genes appear in more than one level in each of the three categories, but no relation between genes is described (apart from them being in the same group). KEGG [8] is one of the most popular pathway databases; it groups genes into pathways of interacting genes and substrates, and contains specific links between genes and substrates that interact directly. Both databases are manually curated but incomplete. The GO database is also redundant, as it contains several terms in the top of the hierarchy that are too broad in their meaning and include thousands of genes. KEGG provides a more detailed organization of the genes but contains information on fewer genes than GO.

Different approaches have been proposed to identify significant gene groups based on lists of differentially expressed genes. Several methods have been implemented that can be directly applied to existing gene-grouping schemes. GOstat [9] compares the occurrences of each GO term in a given list of genes (tested group) with its occurrence in a reference group (typically all the genes on the array) assigning a p value to each term. In the context of pathway analysis, a similar approach is used by Pathway Miner [10], which ranks pathways by p values obtained via a one-sided Fisher exact test. Other methods allow investigators the possibility to define their own gene-grouping schemes. For example, Global Test package [11] applies a generalized linear model to determine if a user-defined group of genes is significantly related to a clinical outcome. With the Gene Set Enrichment Analysis (GSEA) [12,5] an investigator can test if the members of a gene set tend to occur towards the top or the bottom of a ranked gene list obtained from the differential expression analysis, and therefore are correlated with the phenotypic class distinction.

In this paper, we extend the significance analysis of gene pathways to higher order structures, i.e. networks of pathways whose intersections contain a significant number of differentially expressed genes. Network structure can reveal the degree of coordination of different biological functions as a consequence of the treatment, as well as the presence of “focal areas” in which groups of genes play central roles. We show examples in which some biological functions (related to specific pathways) are biologically relevant for the studied process, due to their position inside the pathway network. This analysis can be extended to groups of genes at the “interface” between pathways, whose imbalance can affect more than one biological function.

Our approach is aimed at understanding how external perturbations, such as gene activation or tumor induction, can induce in various types of cells, cell lines or derived tissues, behaviours that can generate, integrate, and respond to dynamic informational cues.

The broad question that we are trying to answer is how a cell converts perturbations to signalling activity into a binary decision resulting from the appearance of a given phenotype. Thus the signalling activity has to be diffused within the cell between and within pathways. A signaling pathway is not a rigid unit, but is made of modules with different functions (e.g. the communication with other pathways) that may be captured by selecting those elements belonging to the interface between pathways.

## Methods

### Data set

The first dataset we consider consist of time course gene expression arrays based on reconstituted c-*myc*^-/-^ rat fibroblast cell lines with the conditionally active, tamoxifen-specific c-Myc-estrogen receptor fusion protein. Binding of Tamoxifen to the estrogen receptor domain elicits a conformational change that allows the fusion protein to migrate to the nucleus and to act as a transcription factor.

This data set (MYC data set) contains the gene expression data collected after the addition of Tamoxifen. Samples were harvested at five time points after the addition of Tamoxifen to the culture medium: 0, 2, 4, 8, and 16 h. The entire experiment was repeated on three separate occasions, providing three biological replicates for each gene and time point. Expression profiling was done by using the Affymetrix platform and U34A Gene Chips [13].

The second dataset we consider consists of the gene expression measurements described in [14]. This data set contains bone marrow samples obtained from acute leukaemia patients, that can be classified as Acute Lymphoblastic Leukemia (ALL) and Acute Myeloid Leukemia (AML). The mRNA prepared from bone marrow mononuclear cells was hybridized with Affymetrix Hgu6800 containing probes for 6817 human genes. The experimental design is a comparison between ALL and AML (one factor) on the basis of 6817 probes. The dataset (AML/ALL dataset) contains 72 samples, 47 obtained from ALL patients and 25 obtained from AML patients.

### Gene selection and pathway grouping

For the MYC dataset, one-way ANOVA was applied to each of the 8799 probe sets to identify those that significantly changed expression level over time. A *p* value of 0.05 was chosen as the cut-off significance level. No post-hoc correction for multiple testing (i.e. Benjamini-Hockberg, FDR) was applied, since post-hoc validation is provided by pathway analysis: 765 genes resulted significant, 251 of which are annotated in KEGG and belonged to 142 pathways.

The AML/ALL dataset was analysed with a linear model with an empirical Bayes method to shrink gene variances (limma, R package) and 1924 genes were found as significantly differentially expressed between the AML and the ALL groups (p <0.05). Among the differentially expressed genes, 801 genes were annotated in the KEGG database.

### Pathway significance and pathway network

In order to reconstruct a network, we need to specify both its nodes and links. From a biological point of view, nodes can be defined as groups of genes (such as pathways or ontologies) coding for proteins/peptides with similar functional properties (e.g. ion channels, kinases, phosphatases, and transcription factors), performing similar tasks or involved in the same biological function. The links between nodes can be drawn in various ways and their definition may also depend on the particular type of experimental design (e.g. temporal correlation or physical interactions of proteins) [15,16].

We choose to define network nodes as groups of genes belonging to the same pathway as described in the KEGG database. To each node we associate a feature corresponding to the state of the pathway, which can be significantly involved (overrepresented), significantly not involved (underrepresented) or not significant in the experimental context [17]. The same classification is used for the links between nodes by analyzing the ratio of significant genes at the intersection between the corresponding pathways.

Significance of nodes and links can be assessed within the framework of 2×2 contingency tables (Table 1) where:

**Table 1:** 2X2 Contingency table.

	Differentially expressed	Not differentially expressed	
∈	α	β	*N*_G_
∉*G*	γ	δ	$N_{\bar{G}}$
	*S*	$\bar{S}$	*N*

α = number of significant genes ∈ *G*, β = number of not significant genes∈ *G*, γ = number of significant genes ∉ *G*, δ = number of not significant ∉ *G*, *S* = α + γ = number of significant genes in the array $\bar{S}=\beta +\delta$ = number of not significant genes in the array, *N_G_* = α + β = number of genes ∈ *G*, $N_{\bar{G}}=\gamma +\delta$ = number of genes ∉ *G*, *N* = total number of measured gene*s*

α = number of significant genes ∈ *G*

β = number of not significant genes ∈ *G*.

γ = number of significant genes ∉ *G*.

δ = number of not significant ∉ *G*.

*S*= α +γ = number of significant genes in the array

$\bar{S}=\beta +\delta$ = number of not significant genes in the array

*N_G_* = α + β = number of genes ∈ *G*

$N_{\bar{G}}=\gamma +\delta$ = number of genes ∉ *G*

*N* = total number of measured gene*s*

Given a subset *G* of the *N* measured genes with *N_G_* genes, α will be differentially expressed while β = *N_G_* - α will not. We compare α and β to the number of differentially expressed genes γ and not differentially expressed genes δ not belonging to *G*. The statistical significance of the contingency table can be computed in different ways: Fisher exact test, binomial and χ^2^ distribution-based tests [17]. We chose to apply the Fisher exact test because the computation of the hypergeometric distribution is straightforward for tables with both small numbers (arising when testing intersections, see below) and large numbers (arising when testing pathways). The Fisher exact test first computes the probability p* of the observed 2×2 table by using the hypergeometric distribution with parameters (*S*,*N_G_*,*N*):

$$p^{*}=p\left(X=\alpha |S,\;G,\;N\right)=\frac{\left(\begin{array}{l} S\\ \alpha \end{array}\right)\left(\begin{array}{l} N-S\\ N_{G}-\alpha \end{array}\right)}{\left(\begin{array}{l} S\\ \alpha \end{array}\right)}=\frac{S!\bar{S}!N_{G}!N_{\bar{G}}!}{\alpha !\beta !\gamma !\delta !N!}$$

The p value to reject the null hypothesis (independence of rows and columns in the contingency table) is given by the sum of the probabilities of all the tables with a probability lower than p* and with the same marginal totals, that is:

$$p=\sum_{\begin{array}{l} i=1,N_{G}\\ pi\leq p^{*} \end{array}}\frac{\left(\begin{array}{c} S\\ i \end{array}\right)\left(\begin{array}{c} N-S\\ N_{G}-i \end{array}\right)}{\left(\begin{array}{c} S\\ i \end{array}\right)}$$

This procedure gives a probability for a two-tailed Fisher test. Distinction between over- or under-representation of the selected group of genes *G* can then be obtained by comparing the proportion α/ *N_G_* of differentially expressed genes in *G* with the proportion of differentially expressed genes *S/N* on the array. A group G is considered significant if *p* ≤ 0.05.

We apply a similar framework to evaluate the significance of all non-empty intersections between two pathways:

$$\begin{array}{cc} {\text{Q}}_{\text{ij}}={\text{G}}_{\text{i}}\;\cap {\text{G}}_{\text{j}}; & \forall \text{i,j}\;=1,...{\text{N;Q}}_{\text{ij}} \end{array}\neq \left\{\emptyset \right\}.$$

The only difference is the definition of the total number of genes *N*, which is taken to be equal to the total number of genes in the two groups. More precisely, for the group intersection significance analysis, the significant genes in the intersection S_I_ and the total number of genes N_I_ in the intersection are compared to the total number of genes found in the union of the two groups and the number of significant ones, *N_G_1_∪G_2__* and *S_G_1_∪G_2__* respectively. The reason for this choice is as follows. Suppose we have two groups with 100 genes each and with 50 genes in common. Suppose 60 genes are significant in each group, 30 of which are in the intersection. If 60% call rate is significant for the two sets, it is likely that it will also be for the intersection. However if we take a random subset of 50 genes from 150 genes in the union of the two groups, we can expect on average a 60% call rate. Hence a random subset with the same numbers of genes as the original intersection would be likely to be significant. By using the union of the two groups as a background, we increase the requirement for the intersection to be considered as significant and reduce the above problem. Intersections are considered significant if their *p* value is lower than 0.05, in which case a link is drawn between the two pathways either red if it is “significantly involved” or blue if it is “significantly not involved”, while if *p* >0.05 no link is drawn.

All the gene groups that we consider are biological pathways defined according to the KEGG annotation, and the mapping between probes and pathways is accomplished by querying the KEGG Database via R software (KEGGSOAP package).

Once the significant links and nodes are established, we perform a meta-analysis on the obtained network structure. The aim of this analysis is twofold: first, the network structure (e.g. the presence of sub-networks, clusters, communities) can reveal important biological features. Second, each network element (node or link) can be ranked not only on the basis of its statistical significance (the *p* value obtained by the above method) but also considering its *centrality* in the network. We consider as a centrality measure the Betweenness Centrality (BC) for each vertex, a parameter that characterizes the degree of “trafficking” through a network element [18]. For a given vertex, BC is proportional to the sum of the shortest paths passing through it:

For a graph G with *n* nodes, the Betweenness Centrality BC (*v*) for node *v* is

$$\text{BC}\left(\nu \right)=\sum_{s\neq \nu \neq t}\frac{\sigma_{st}\left(\nu \right)}{\sigma_{st}}$$

Where σ_*st*_ is the number of shortest paths from node *s* to *t*, and σ_*st*_(*v*) is the number of shortest paths from *s* to *t* that pass through a node *v*.

### Results

The list of pathways obtained from the Fisher test shows the most significant overrepresented and underrepresented pathways in the MYC (Table 2) and AML/ALL datasets (Table 3). In the MYC dataset three pathways are significantly underrepresented: neuroactive ligand receptor interaction, Cytokine-Cytokine receptor interaction, and Jak-STAT signalling pathway. Among the overrepresented pathways are RNA and DNA polymerase, Cell cycle and some metabolic and bio-synthetic pathways (Pyrimidine, fatty acid and Ether lipid metabolism, folate and Glycan structures Biosynthesis). In the case of AML/ALL dataset two pathways are found to be underrepresented (Ribosome and Neuroactive ligand receptor interaction); among the overrepresented pathway are Cell Cycle, many metabolic pathways (Glycerophospholipid, Galactose, Pyrimidine and Purine metabolism among other) and a signaling pathway (B cell receptor signaling pathway).

**Table 2:** MYC dataset: statistically significant pathways. The table shows the significantly over- or under-represented pathways (*p* < 0.05) for the MYC dataset. For each pathway the *p* value, the total number of genes, the number of significant genes and the over or under-representation status are shown.

Pathway	*p* value	genes	Significantgenes	Under/Over representation
Neuroactive ligand-receptor interaction	1.02E-05	223	11	under
RNA polymerase	0.000354	4	4	over
Cytokine-cytokine receptor interaction	0.002103	87	3	under
Pyrimidine metabolism	0.002192	23	9	over
DNA polymerase	0.003769	9	5	over
Aminophosphonate metabolism	0.009321	4	3	over
Cell cycle	0.014101	44	12	over
N-Glycan biosynthesis	0.016643	12	5	over
Jak-STAT signaling pathway	0.019052	69	3	under
Folate biosynthesis	0.025244	9	4	over
Fatty acid metabolism	0.027632	37	10	over
Ether lipid metabolism	0.044526	15	5	over
Glycan structures - biosynthesis 1	0.046622	20	6	over

**Table 3:** AML/ALL dataset: statistically significant pathways. The table shows the significantly over- or under-represented pathways (*p* < 0.05) for the AML/ALL dataset. For each pathway the *p* value, the total number of genes, the number of significant genes and the over or under-representation status are shown.

Pathway	*p* value	genes	Significant genes	Under/Over representation
Ribosome	6.70E-05	78	10	under
Cell cycle	0.00152	80	40	over
Glycerophospholipid metabolism	0.003631	28	17	over
Neuroactive ligand-receptor interaction	0.004484	211	51	under
Galactose metabolism	0.008409	21	13	over
B cell receptor signaling pathway	0.008725	49	25	over
Aminoacyl-tRNA biosynthesis	0.015056	20	12	over
Pyrimidine metabolism	0.016126	51	25	over
Purine metabolism	0.02202	90	40	over
Glycerolipid metabolism	0.024062	39	20	over
Leukocyte transendothelial migration	0.0245	75	34	over
Histidine metabolism	0.033768	26	14	over
Nitrobenzene degradation	0.03577	3	3	over
Proteasome	0.040852	27	14	over
Reductive carboxylate cycle (CO2 fixation)	0.043013	7	5	over
Protein export	0.043013	7	5	over
Aminophosphonate metabolism	0.043363	5	4	over
Nucleotide sugars metabolism	0.043363	5	4	over

**Figure 1:**
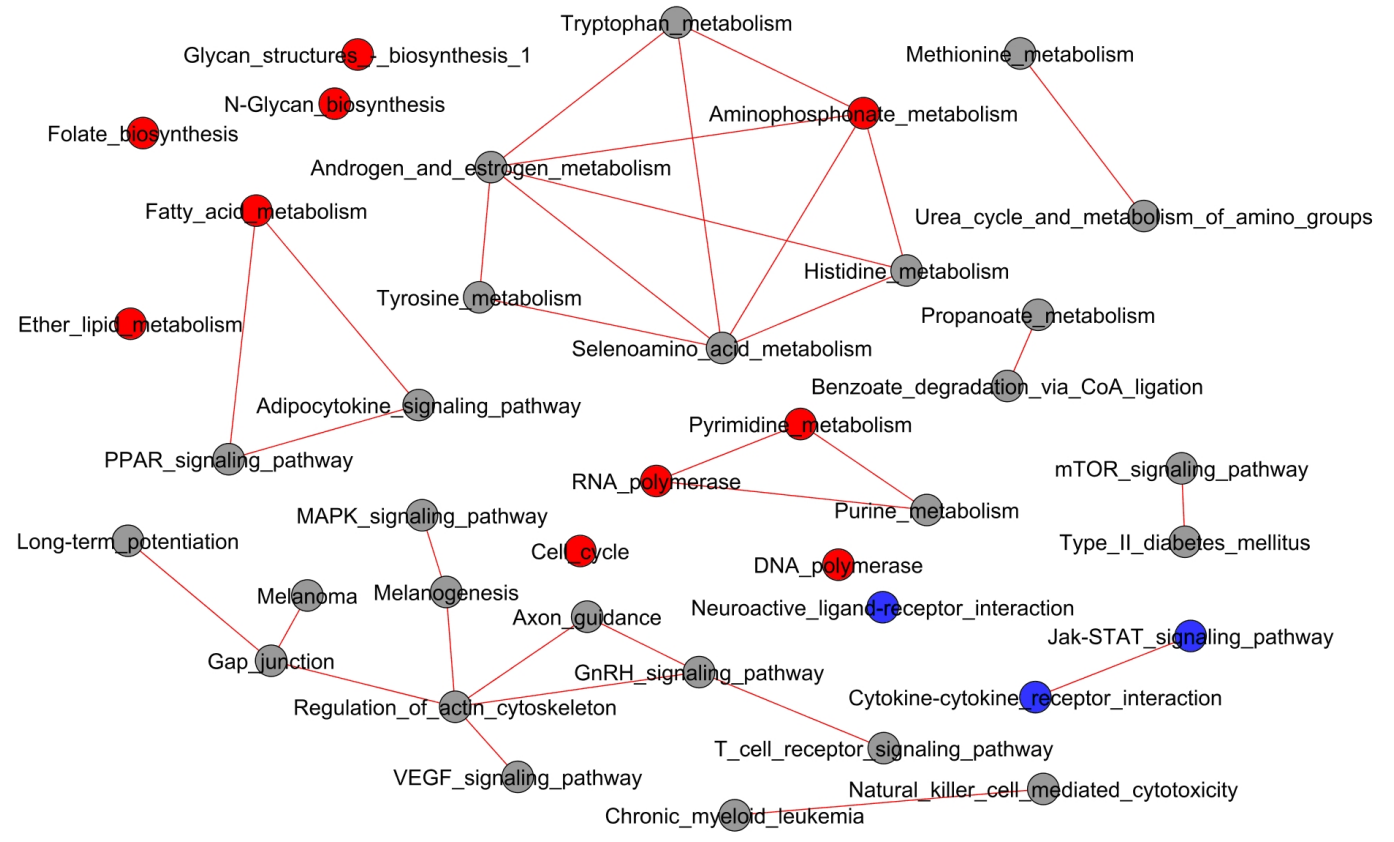
**Network of pathways for MYC dataset**. Gray circles indicate not significant pathways, red and blue circles indicate significant pathways that are respectively overrepresented and underrepresented. Red links indicate pathways interconnections that resulted statistically significant and overrepresented. The largest pathway sub-networks are clearly related to Metabolism, Genetic Information Processing and Signalling biological functions.

**Figure 2:**
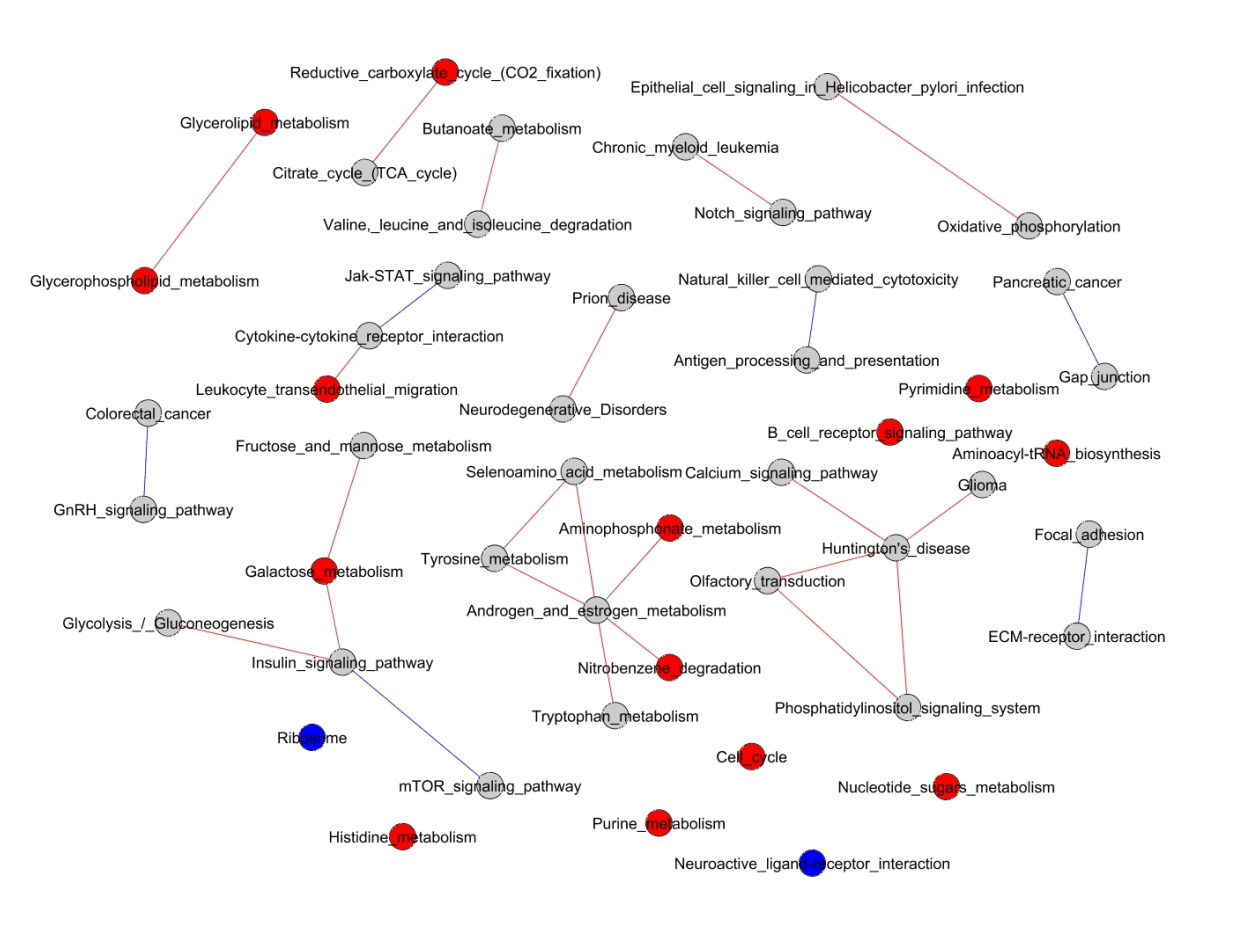
**Network of pathways for the AML/ALL dataset**. Gray circles indicate not significant pathways, red and blue circles indicate significant pathways that are respectively overrepresented and underrepresented. Red lines indicate pathways interconnections (links) that resulted statistically significant and overrepresented, whereas blue lines indicate significant pathways interconnections that are underrepresented.

In our case studies (see Figures 1, 2) the networks are very small, due to the sparseness of the significant links and nodes, thus very few network elements have nontrivial values of BC. Anyway, the analysis of the MYC network shows the emergence of four main sub-networks (Figure 1). These sub-networks are related to different biological functions: the first sub-network is composed by pathways involved in signalling processes (MAP Kinases Signalling Pathway, VEGF Signalling Pathway, Gonadotropin-releasing hormone (GnRH) Signalling Pathway) and pathways that are related to the communication between cells and the external environment (Regulation of actin cytoskeleton and Gap junction). Another interesting sub-network connects the Metabolism with the Signalling system, showing links between PPAR Signalling Pathway, Adipocytokine Signalling Pathway and Fatty Acid Metabolism. A further sub-network is related to nucleic acids precursors synthesis and nucleic acids polymerisation (Pyrimidine metabolism, RNA polymerase and Purine metabolism). Another interesting sub-network contains some basic metabolic pathways (Aminophosphonate, Tryptophan and Tyrosine metabolism androgen and estrogen metabolism among them).

For the AML/ALL dataset, the pathways network showed in Figure 2 evidences a sub-network connecting signalling and metabolism (Insulin Signalling pathway, Glycolysis/ Gluconeogenesis, Galactose Metabolism, Fructose and Mannose Metabolism and mTOR Signalling Pathway that shows an underrepresented intersection with Insulin Signalling Pathway). Another interesting sub-network contains some basic metabolic pathways (Aminophosphonate, Tryptophan and Tyrosine metabolism androgen and estrogen metabolism, etc.). The presence of a sub-network involving Calcium and Phosphatidylinositol signalling, Huntington disease, Glioma and olfactory transduction can be noticed.

As far as BC is concerned, the most central pathway for the MYC dataset is Regulation of Actin Cytoskeleton followed by Gap Junction and GnRH signalling pathway (Table 4). For the AML/ALL dataset, the most central pathway is the Androgen and Estrogen metabolism followed by Huntington disease and Insulin signalling pathway (Table 5).

**Table 4:** Pathways ranked according to BC for the MYC dataset. List of pathways showing highest values of BC for the MYC dataset: connectivity degree and node BC are shown for each pathway.

Pathway	Connectivity degree	Node BC
Regulation of actin cytoskeleton	5	29
Gap junction	3	15
GnRH signaling pathway	3	8
Melanogenesis	2	8
Seleno aminoacid metabolism	5	1.833
Androgen and estrogen metabolism	5	1.833
Aminophosphonate metabolism	4	0.333

**Table 5:** Pathways ranked according to BC for the AML/ALL dataset. List of pathways showing highest values of BC for the AML/ALL dataset: connectivity degree and node BC are shown for each pathway.

Pathway	Connectivity degree	Node BC
Androgen and estrogen metabolism	5	9
Huntington disease	4	5
Insulin signaling pathway	3	5
Galactose metabolism	2	3
Cytokine-cytokine receptor interaction	2	1

**Figure 3:**
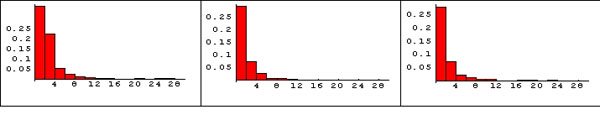
**Distribution of gene pathway membership for MYC dataset**. The y axis is the gene frequency and the x axis is the number of pathways that genes belong to. The majority of genes belong to few pathways whereas few genes (hubs) belong to several pathways (scale free distribution). A) Histogram obtained from the KEGG database for the Rattus norvegicus organism. B) Histogram obtained from the genes on rat Affymetrix U34A Gene Chip. 3) Histogram obtained from the significant genes for MYC dataset.

The existence of important genes belonging to the interface among pathways for the MYC dataset is clearly evidenced in (Figure 3) where the distribution of pathway membership (the number of pathways that a gene belongs to) is showed for all the genes obtained from Rattus norvegicus KEGG database, for the genes on the rat Affymetrix U34A Gene Chip and for the significant genes arising from c-Myc activation. All the three histograms show the same heavy-tailed distribution, meaning that the majority of genes belong to few pathways whereas few genes (the hubs) belong to several pathways.

**Figure 4:**
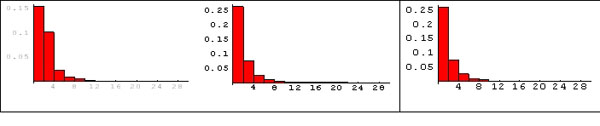
**Distribution of gene pathways membership for AML/ALL dataset**. The y axis is the gene frequency and the x axis is the number of pathways, as in Fig 3. A) Histogram obtained from the Homo sapiens KEGG database. B) Histogram obtained from the genes on Affymetrix Hgu6800 array. 3) Histogram obtained from the significant genes from AML/ALL dataset.

In (Figure 4) the pathway membership distributions for the AML/ALL dataset are shown and similar results are obtained. Histograms from Homo sapiens KEGG database, for the whole Affymetrix Hgu6800 array genes and for the selected genes, are of the heavy-tailed type, highlighting the presence of hub genes.

**Figure 5:**
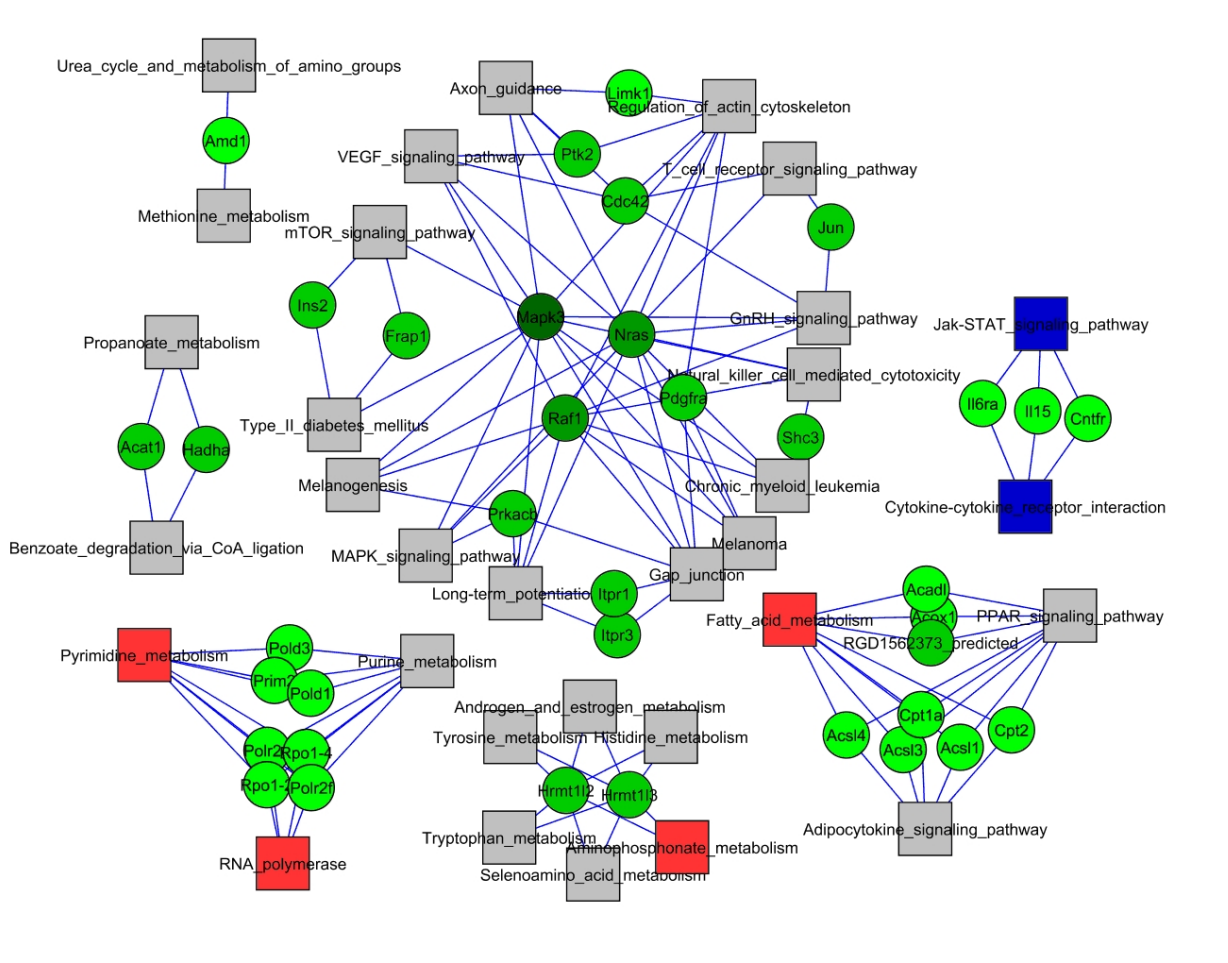
**Bipartite graph of pathways and genes for the MYC dataset**. Pathways are represented by squares and genes by circles. The isolated pathways have been removed so that the graph contains only the connected components of Figure 1, evidencing only the significant genes of the significant intersections. Red and blue squares indicate respectively significantly overrepresented and underrepresented pathways whereas the gray indicates not significant pathways. The green tone indicates the degree of pathways membership: from light green (connected to few pathways) to dark green (the hubs).

The bipartite graphs highlight the central role of hub genes emerging from the pathway networks. In the MYC dataset (Figure 5) the Signalling sub-network is strongly connected and presumably coordinated by a small number of genes such as MAP Kinase III (*Mapk3*), neuroblastoma ras oncogene (*Nras*), v-raf-1 murine leukemia viral oncogene homolog 1 (*Raf1*), platelet derived growth factor receptor alpha polypeptide (*Pdgfra*) and cell division cycle 42 homolog (*Cdc42*). The sub-network containing the basic metabolic pathways (Aminophosphonate, Tryptophan and Tyrosine metabolism, androgen and estrogen metabolism, etc.) shows at its intersections genes belonging to the family of protein arginine methyltransferases (*Hrmt1l2*, *Hrmt1l3*) that are involved in histone modification and chromatin remodelling.

**Figure 6:**
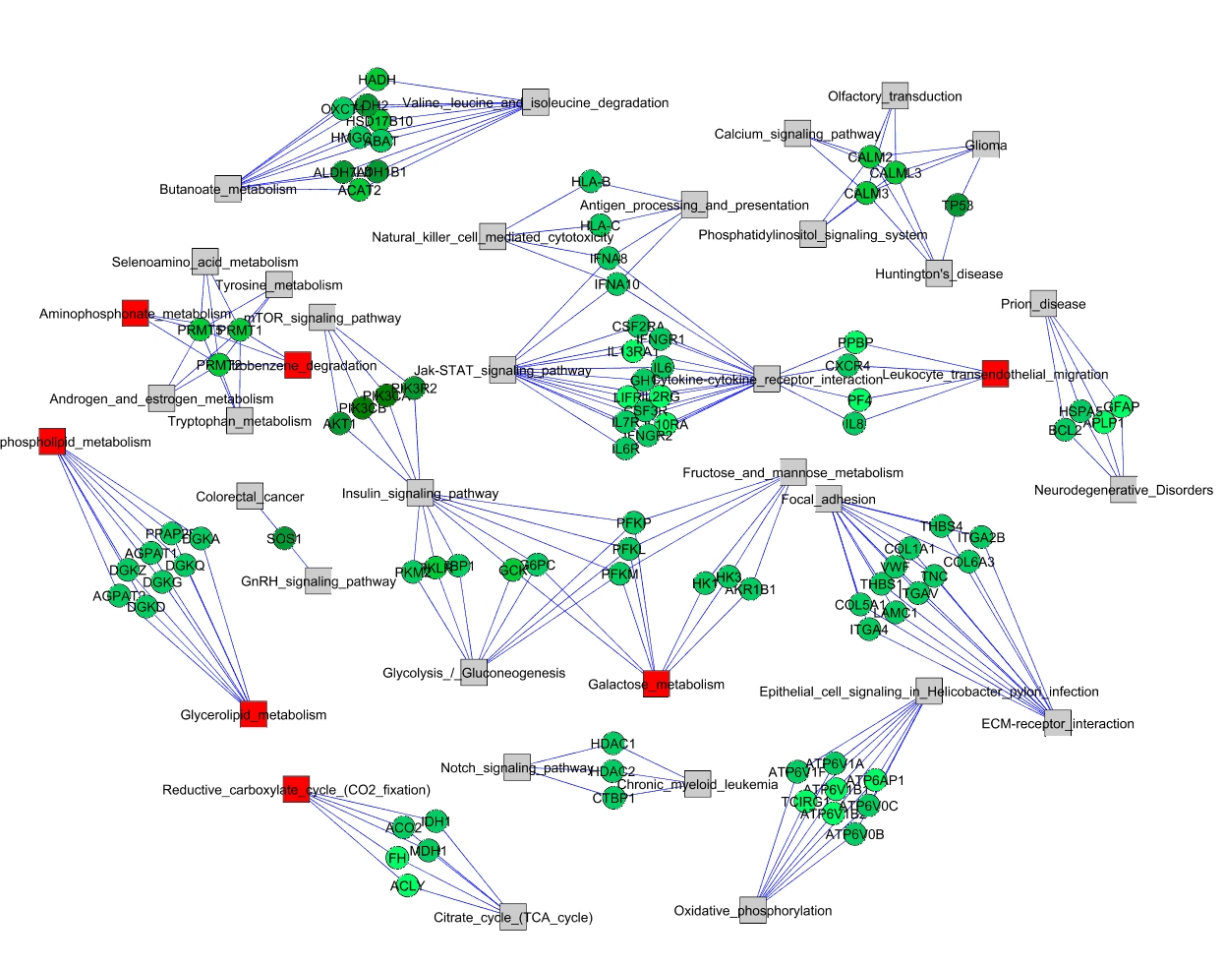
**Bipartite graph of pathways and genes for the AML/ALL dataset**. Pathways are represented by squares and genes by circles. The isolated pathways have been removed so that the graph contains only the connected components of Figure 2, evidencing only the significant genes of the significant intersections. Red and blue squares indicate respectively significantly overrepresented and underrepresented pathways whereas the gray indicates not significant pathways. The green tone indicates the degree of pathways membership: from light green (connected to few pathways) to dark green (the hubs).

As far as the AML/ALL dataset (Figure 6) is concerned, in the sub-network connecting Signalling and Metabolism, especially at the intersection between insulin and mTOR signalling pathways, some crucial genes emerge (*PIK3CA*, *PIK3CB*, *PIK3R2* and *AKT1*).

Similarly to the MYC dataset, the sub-network that contains some basic metabolic pathways (Aminophosphonate, Tryptophan and Tyrosine metabolism, androgen and estrogen metabolism, etc.) shows at their intersection genes belonging to the family of protein arginine methyltransferases (*PRMT1* that is homologue to the rat counterpart *Hrmt112* seen in the MYC dataset, *PRMT2* and *PRMT5*).

It is worth noticing that in the sub-network involving both Calcium and Phosphatidylinositol signalling it is possible to evidence some crucial genes, for example calmodulin and the well known tumor protein p53 (*TP53*).

## Discussion

A global picture of gene expression is greatly enhanced by the use of genes categorization and pathway analysis, but there can be several cases where this approach is not completely satisfactory. It may fail to capture the relationship between the categories and it may discard some important pathways or genes, because it does not take into account their relevance based on their central position. A typical case is that of genes that are at the interface among pathways (as in the case of hubs). With our method we try to overcome these limitations assigning more relevance to the position occupied by a group of genes in a higher level structure (i.e. the pathway network) in addition to their statistical significance alone.

The comparison between the pathways listed in Table 2 and Table 3 with the corresponding networks of Figure 1 and Figure 2 shows how the lists alone can not grasp the complexity of pathway activation induced by gene expression changes, remarkably most of the pathways in these networks are not listed in Table 2 and 3.

For the MYC dataset, the biggest sub-network comprises pathways that are involved in signalling processes (MAP Kinases Signalling Pathway, VEGF Signalling Pathway, GnRH Signalling Pathway), and in structural reorganization, communication and connections (Regulation of actin cytoskeleton and Gap junction). The bipartite graph shows how this sub-network is strongly connected and coordinated by a small number of hub genes (*Mapk3*, *Nras*, *Raf1*, *Pdgfra*, *Cdc42*). All these genes are well-known proto-oncogenes involved in proliferation, regulation of growth, cell cycle progression control and structural reorganization of the cell. They are major c-Myc downstream effectors and they are responsible of the profound effects that c-Myc exerts in cellular physiology: the down-regulation of the connections among cells and the connections between cells and the extracellular matrix, cytoskeleton reorganization, and the induction of cell growth and proliferation [19]. The MYC network evidences also a strongly connected component related to basic metabolism, comprising both biosynthetic and catabolic pathways and a small sub-network related to the synthesis of nucleic acids, which are known to be major targets of c-Myc that upregulates both energy metabolism and biosynthesis needed for growth and proliferation [13,19].

For the AML/ALL dataset, the pathways network showed in Figure 2 evidences a sub-network connecting signalling and metabolism that underlines how the regulation of energy metabolism may play a key role in the discrimination between the two types of leukaemia. It is interesting to note that Insulin and mTOR signalling pathways are known to be involved in AML [20,21], in particular the crucial genes (*PIK3CA*, *PIK3CB*, *PIK3R2* and *AKT1*, Fig 6) in the intersection between these two pathways have been recently pointed out as promising novel targets for AML therapy [22].

Among the relevant genes extracted by our method we can notice the well known tumor protein p53 (*TP53*), involved in a wide variety of cancers, found in the sub-network involving Calcium and Phosphatidylinositol signalling systems.

In both datasets the sub-networks containing basic metabolic pathways (Aminophosphonate, Tryptophan and Tyrosine metabolism, androgen and estrogen metabolism, etc.) show at their intersections genes belonging to the family of protein arginine methyltransferases that are involved in histone modification and chromatin remodelling. They have been recently pointed out to have a major role in lymphoid tumours, leukaemia and more generally in cancer [23,24].

The role of epigenetic modification in cancer induction and differentiation, is gaining several experimental evidences and is giving new perspectives on cellular complex processes: our results can also suggest another mechanism for MYC in promoting oncogenesis through chromatin remodelling.

## Conclusion

Our results show that it is possible to combine high-throughput experimental procedures and advanced data processing as a general Systems Biology approach to discover pathway network changes following variation of cellular phenotypes. The use of known pathways, such as those described in the KEGG database, is motivated by the clarity of their biological interpretation, but our method can be applied also to custom defined pathways or to group of genes obtained from other methods. This approach can be further generalized by considering different statistical methods for assessing single gene significance, or the significance of single network modules.

This approach leads to an increased biological insight of the results by adding topological information (Betweenness Centrality) to a list of pathways obtained by significance test. It may improve the comparability of microarray studies, both between different cell types and different perturbations by considering changes in pathway networks instead of single genes.

Moreover, this network-based method highlights the existence of “focal areas” or hub genes that are more likely found in the intersection between pathways. In this way it is possible to reconsider genes on the basis of their central role in the network and not only for their statistical significance. This can be of great importance also considering that the most central genes typically are subjected to very small changes that could be hardly detectable by any single gene statistical analysis, but can anyway exert great biological effects due to their central role in pathway interconnections and communication. Recently other authors are developing methods trying to extract from the data genes that can be biologically relevant even if they are not top-ranking in terms of statistical significance [25].

The problem of assessing pathway relevance is issued in a different way by Draghici [26]. In his paper the biological relevance of each pathway is scored both on the basis of a statistical significance test (pathway enrichment analysis) and on other parameters referred to the position of single genes in the pathway. Our method for pathway relevance can be seen as a top-down approach (from a KEGG-based network to single pathways and genes), as much as the method by Draghici is a bottom-up one (from genes to pathways).

## List of abbreviations used

GO: gene ontology, KEGG Kyoto Encyclopedia of Genes and Genomes, GSEA: Gene Set Enrichment Analysis, ALL: Acute Lymphoid Leukemia, AML: Acute Myeloid Leukemia, FDR: False Discovery Rate, ANOVA: Analysis of Variance, BC: Betweenness Centrality.

## Competing interests

The authors declare that they have no competing interests.

## Authors' contributions

MF implemented the algorithm and contributed to writing the manuscript, DR and GC conceived of the study, participated in its design and coordination and helped to draft the manuscript, NN performed dataset acquisition and processing and contributed to writing the manuscript, LNC and JS: interpretation of MYC data and general manuscript revision, EV and LM: interpretation of AML/ALL data and general manuscript revision
